# Supplementary material for: NFC-enabled sensing platform for the onsite determination of asparagine in food
Source: Mater Today Bio. 2025 Dec 12;36:102675. doi: 10.1016/j.mtbio.2025.102675 (PMC12774780; doi:10.1016/j.mtbio.2025.102675)
Supplement: Multimedia component 1 [file mmc1.docx]

Supporting Information

**NFC-enabled Sensing Platform for the Onsite Determination of Asparagine in Food**

Hong Seok Lee ^1^, Ravleen Kaur Panesar ^1^, Laura Gonzalez-Macia ^1^, Giandrin Barandun ^1,2^, and Firat Güder ^1,3^*

^1^ Department of Bioengineering, Imperial College London, SW7 2AZ, United Kingdom

^2^ BlakBear Ltd, 185 Tower Bridge Rd, London SE1 2UF, United Kingdom

^3^ Bezos Centre for Sustainable Protein, Imperial College London, London, SW7 2AZ, United Kingdom

*Corresponding Author: [guder@ic.ac.uk](mailto:guder@ic.ac.uk)


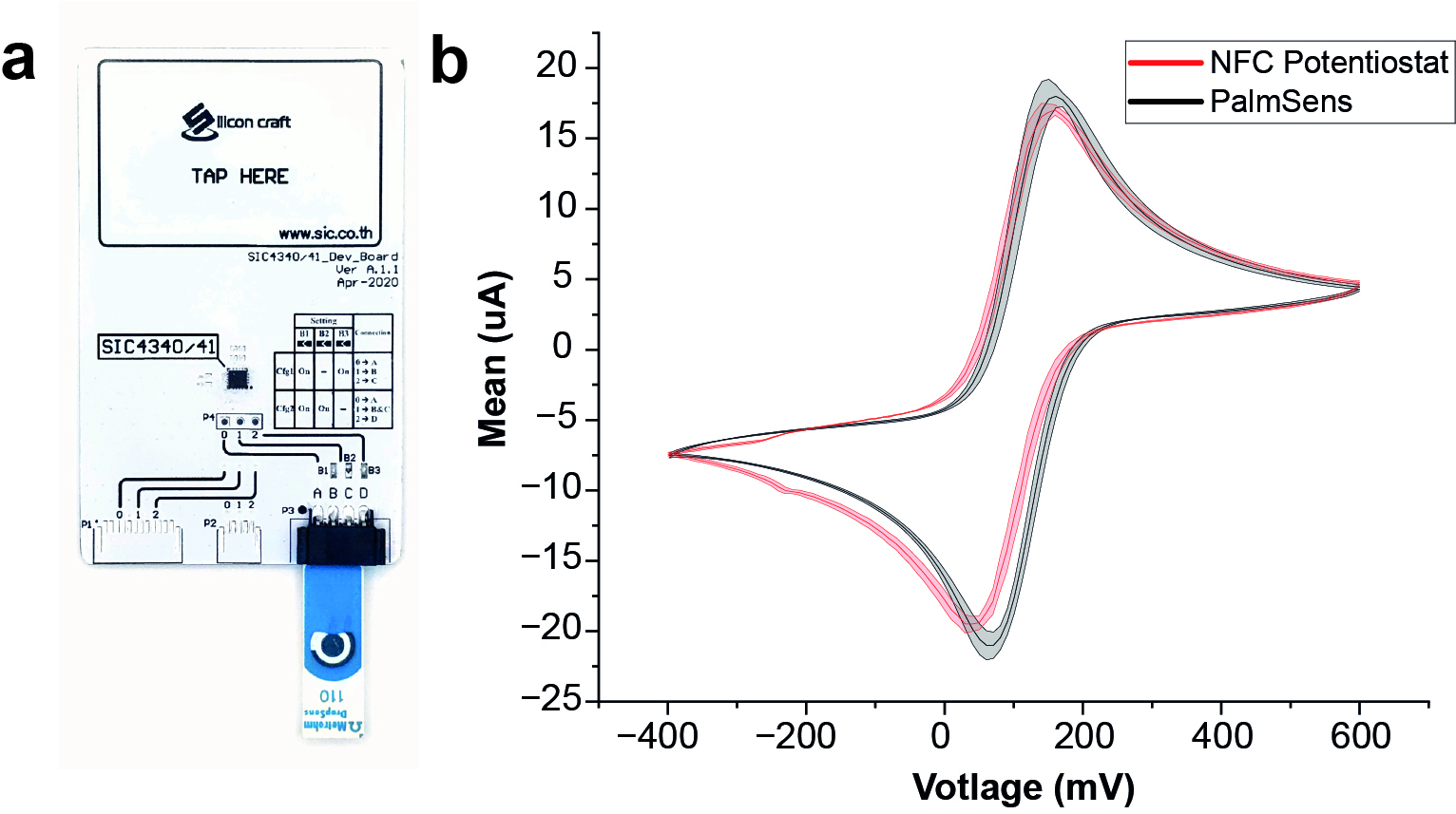


**Figure S1**. **(a)** Photograph of the single chip NFC-based potentiostat **(b)** Cyclic voltammogram comparison of the benchtop commercial potentiostat (PalmSens) to the NFC-based single chip potentiostat using1mM Potassium Ferricyanide in 0.1M KCl at 100mV/s (n = 5)


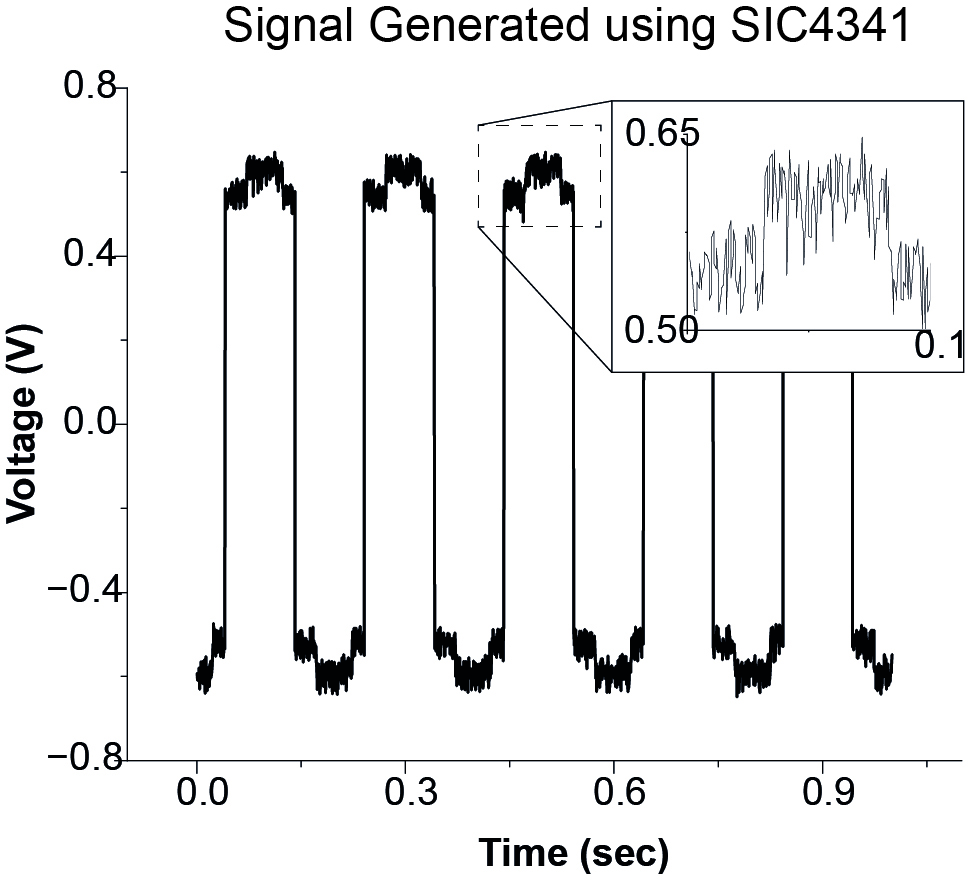


**Figure S2**. Generation of a 5Hz, 1.2V peak-to-peak square wave using the SIC4341 IC, which was commanded by the developed smartphone app. The chip applied an alternating potential (swinging between +0.6V and -0.6V) to the electrodes every 100ms. The resulting signal was applied to the chemPEGS.


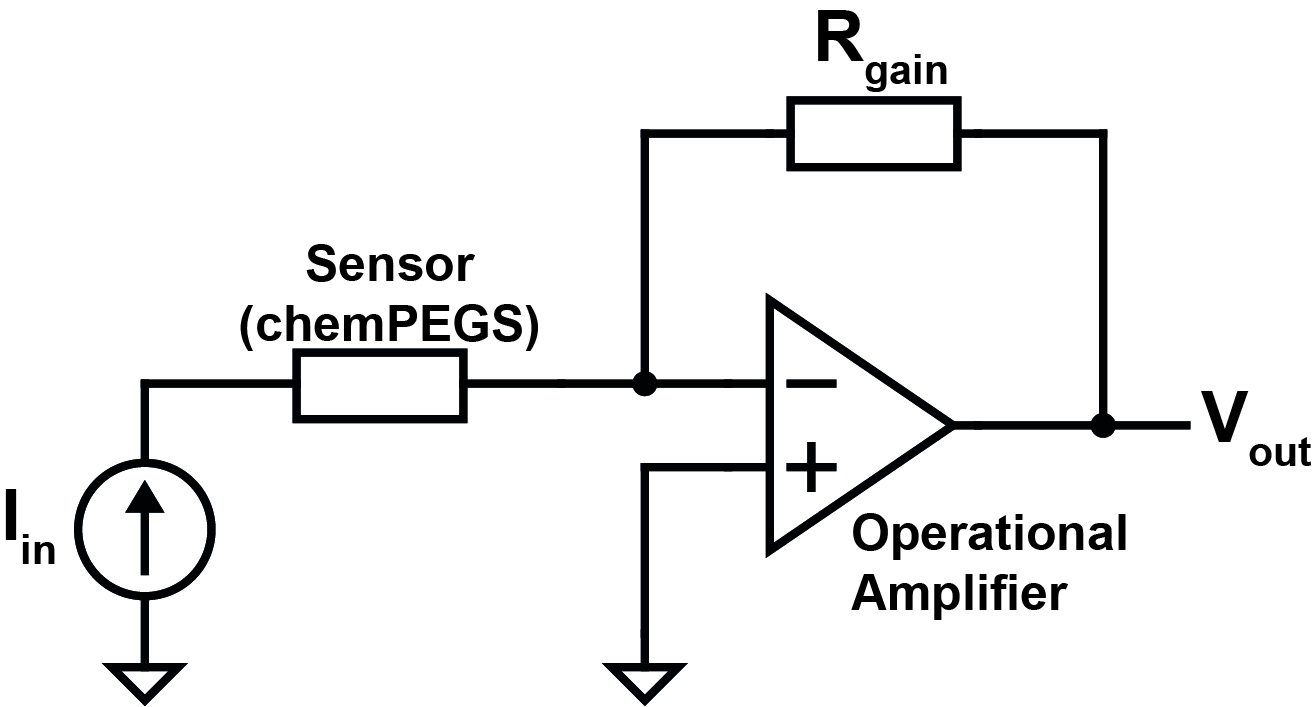


**Figure S3**. To measure the ionic impedance of the chemPEGS, a transimpedance amplifier converts the sensor's current (I_in_) into a proportional voltage (V_out_). The amplification factor is set by the gain resistor (R_gain_), following the relationship:

V_out_ = – R_gain_ × I_in_

The resulting analog voltage (V_out_) is read and digitized by the built-in ADC of the SIC4341 IC or the Arduino Due.


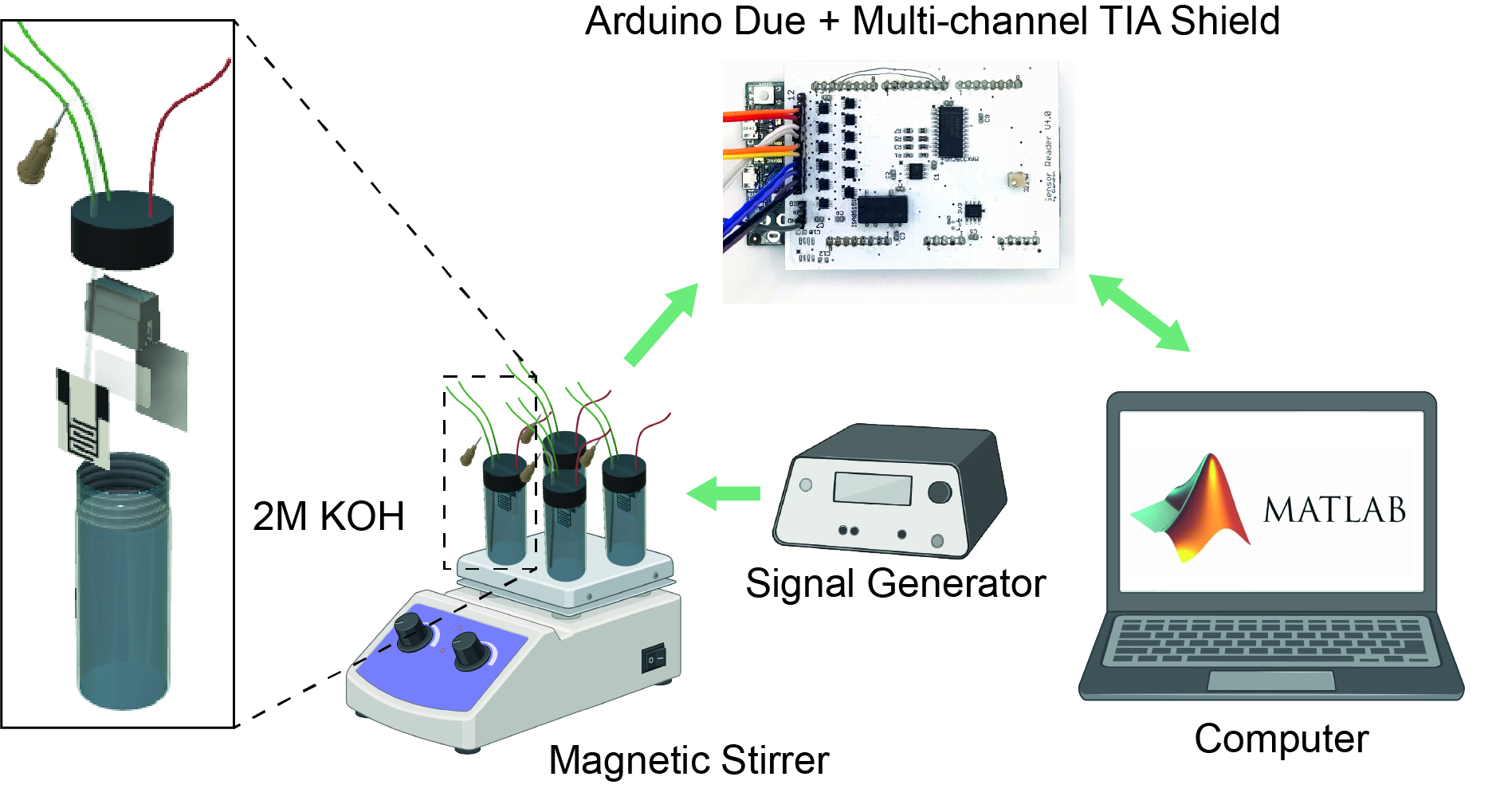


**Figure S4**. Setup for the sensor characterization experiment. Two chemPEGS were place on each glass vial and they were connected by an electrical feedthrough. The sensor signal is amplified and processed by the custom designed multi-channel transimpedance amplifier shield. The shield was connected to the Arduino Due for read-out and plotted on a computer through MATLAB.

**Calculation of rate of change in conductance of chemPEGS**

The rate of conductance change for the chemPEGS sensor was determined through a linear regression analysis performed on a specific segment of the data. To ensure consistency across experiments, the raw conductance data was first normalized. A baseline conductance (G_0_​) was established by measuring the stable conductance of chemPEGS under fully humidified conditions where the liquid-vapor system was in equilibrium. All subsequent conductance measurements were then normalized relative to this G_0_​ baseline.

A 3-minute analysis window was manually selected from a plot of the normalized conductance versus time. This window begins a minute after the introduction of potassium hydroxide (KOH) to the sample solution. This specific interval was chosen because it corresponds to the period where the conductance changes at a constant rate. This change is driven by the neutralization of sulfuric acid on the sensor surface upon exposure to ammonia gas, which is liberated from the enzymatic breakdown of free asparagine. The slope derived from the linear regression of this 3-minute data segment represents the final rate of conductance change correlated to the free asparagine level in the sample solution.

**Linear Regression Analysis.** A linear regression model was applied to the normalized conductance data within selected time interval window. The analysis uses the standard linear equation ^1^:

$$\boldsymbol{y=a+bx}$$

Where,

**y** represents the normalized conductance of the chemPEGS.

**x** represents time.

**b** is the slope of the line, which directly quantifies the rate of change in conductance.

**a** is the y-intercept

The rate of change in conductance, **b** is calculated using the method of least squares.

Mathematically, **b** is calculated with the following formula ^2^:

$$\boldsymbol{b}=\frac{\sum_{i=1}^{n} x_{i}y_{i}-\frac{\left( \sum_{i=1}^{n} y_{i} \right)\left( \sum_{i=1}^{n} x_{i} \right)}{n}}{\sum_{i=1}^{n} x_{i}^{2}-\frac{\left( \sum_{i=1}^{n} x_{i} \right)^{2}}{n}}$$

Where:

- xᵢ and yᵢ are the individual time and conductance data points.
- x̄ and ȳ are the mean of the time and conductance values respectively

This calculation is performed automatically by regression functions of MATLAB to determine the precise rate of change in conductance of chemPEGS.


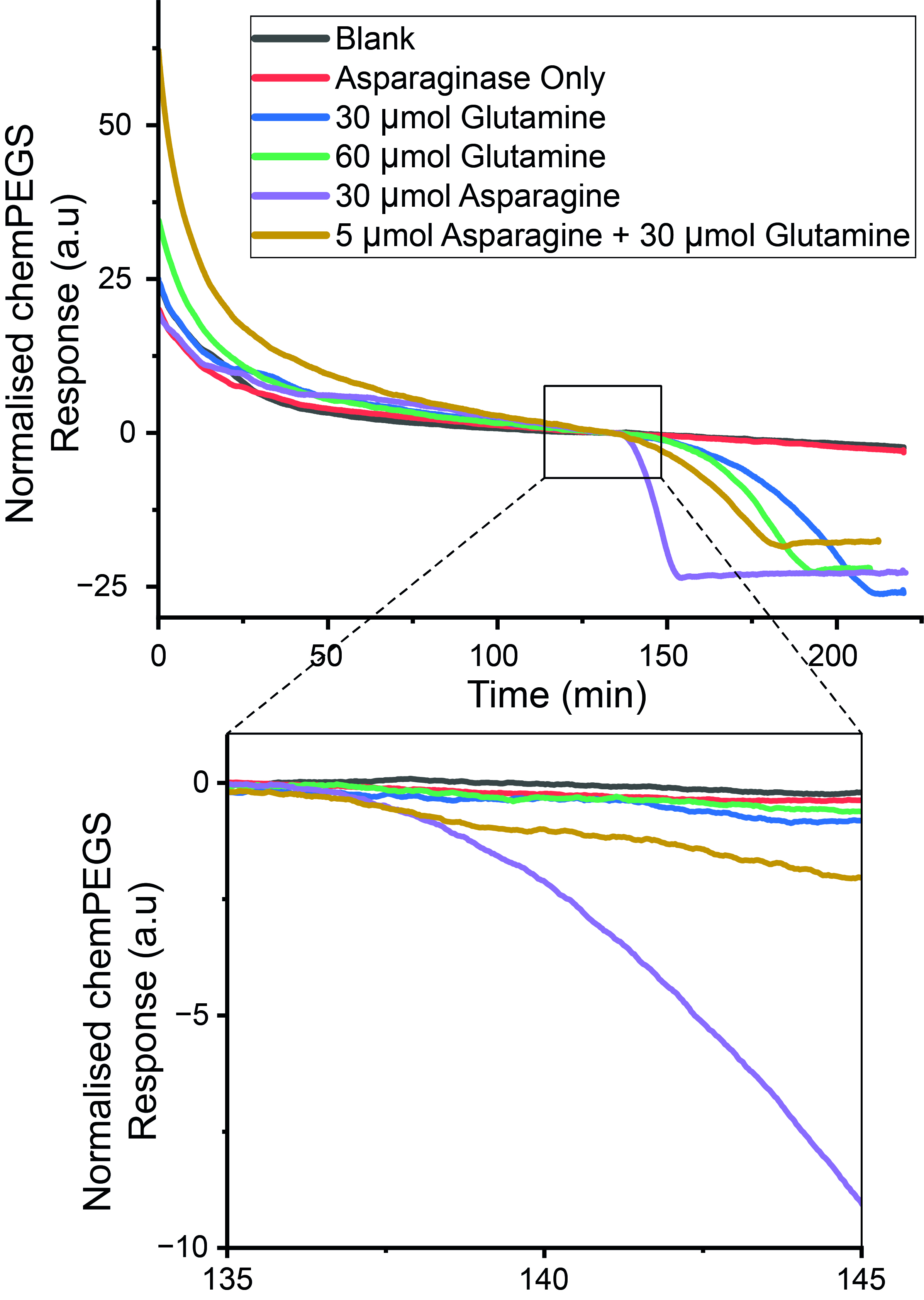


**Figure S5. chemPEGS response to Asparaginase, L-asparagine and L-glutamine.** Asparaginase was introduced to all samples (excluding the blank matrix) at the start of the experiment. At the 135-minute mark, once the chemPEGS sensor had fully humidified and achieved stable conductance, 2M KOH was added. Although strong alkaline conditions can induce L-glutamine hydrolysis, the kinetics of this reaction are slow^3^.

Glutamine + OH^-^ => Glutamate + NH_3_

Consequently, the signal contribution from L-glutamine is negligible during the first 10 minutes. Therefore, the initial chemPEGS response is dominated by the volatilization of free ammonia produced by the enzymatic conversion of asparagine. Significant interference from amide hydrolysis was observed only after 10 minutes.

| Component | Cost (USD) |
| --- | --- |
| **Reusable Hardware (One-Time Cost)** | |
| 3D Printed Mini Magnetic Stirrer (Electronics + Hardware components) | $ 7.50 |
| Glass Vial + Lid | $ 2.52 |
| NFC PCB | $ 0.04 |
| SIC4341 IC Chip | $ 0.10 |
| Capacitors (x2) | $ 0.02 |
| 3mL Leur-lock Syringe (x2) | $ 0.31 |
| Leur-lock Tip | $ 0.06 |
| 10cm Silicone Tube | $ 0.18 |
| **Total** | **$ 10.73** |
|  |  |
| **Consumable Components (Per-Test Cost)** | |
| L-Asparaginase | $ 1.26 |
| Potassium Hydroxide (KOH) | $ 0.11 |
| Sulfuric Acid (H_2_SO_4_) | <$ 0.01 |
| Paper-based Electrical Gas Sensor | <$ 0.01 |
| **Total** | **$ 1.39** |

**Figure S6**. The cost breakdown of the NFC-based FAsn sensing system

**Reference**

1. The MathWorks Inc. Perform regression with deep learning neural networks. *MathWorks Help Center* https://uk.mathworks.com/help/deeplearning/ref/regression.html.

2. Douglas C. Montgomery & George C. Runger. *Applied Statistics and Probability for Engineers*. Wiley (**2018**).

3. Arii, K., Kobayashi, H., Kai, T. & Kokuba, Y. Degradation kinetics of l-glutamine in aqueous solution. *Eur. J. Pharm. Sci.* **9**, 75–78 (**1999**).
